# Speech of Hon. N. J. Hammond, LL.D.

**Published:** 1899-05

**Authors:** 


ATLANTA

### Journal-Record of Medicine.

Successor to Atlanta Medical and Surgical Journal, Established 1855, and Southern Medical Record, Established 1870.

Vol. I. MAY, 1899. No. 2.

BERNARD WOLFF, M.D., DUNBAR ROY, A.B., M.D., } EDITORS.

M. B. HUTCHINS, M.D., BUSINESS MANAGER.

### ORIGINAL COMMUNICATIONS.

### SPEECH OF HON. N. J. HAMMOND, LL.D.*

At the Annual Commencement of the Atlanta College of Physicians and Surgeons, Grand Opera House, April 3, 1899.

*Members of the Board of Trustees, Doctors, and Ladies and Gentlemen:*

The Atlanta College of Physicians and Surgeons comes from the lately consummated legal union of the Atlanta Medical College and the Southern Medical College, under the amended charter of the former. Therefore its life may be regarded as having begun on February 14, 1854, the date of that charter. The original trustees and incorporators were L. C. Simpson, Jared I. Whitaker, John Collier, John L. Harris, Green B. Haygood, James M. Calhoun, Hubbard Cozart, Daniel Hook, and William Herring.

*Since this address was delivered Col. Hammond has died. This was his last public speech, and is a fitting monument to his sterling ability, since it has been pronounced by able critics to be the most finished address ever heard delivered at a medical college commencement.

In December, 1857, Georgia donated to the trustees $15,000.00, to enable them to complete the college and furnish it with a museum and library, etc., in consideration of which one student from each congressional district of Georgia, selected by its Representative in Congress, was to be allowed to attend each course of lectures in said college free of charge.

Under the original charter, the trustees were authorized to grant no degree but that of Doctor of Medicine. It may and does now grant also degrees in Dental Surgery and Pharmacy.

The present Board of Trustees is composed of twenty-one members, chosen from the boards of the two colleges lately united. The Southern Medical College was twenty years old, and brought into this union all which those later years enabled earnest, well-trained and deeply interested doctors to accumulate for such purposes. The buildings, the museums, libraries and other property of the two colleges united give us an institution unequaled by any south of Baltimore, and which offers to students as full opportunities as are to be had anywhere at so small expense, in this exceptionally good climate, and with such energy in business affairs in this city as to impart a valuable object-lesson to the young men coming within our influence. Here, too, they are trained mostly with Georgians, who throughout life will be their fellow-citizens and cooperators in professional work.

All this seems proper for this its first commencement, that you may know the origin, growth, and accession to the new college, which has for the past year had three hundred and thirty-one students, including the Dental Department, and has graduated this year in Dentistry thirty-one, in Pharmacy thirteen, and in Medicine fifty-four, and given to them their respective degrees.

To one who has lived here ever since December, 1853, the recalling of these long past facts presents a strong temptation to speak of Atlanta as it was, contrasted with what it is; when, for instance, the post-office closed from 12 o'clock M. to 2 P.M., and from 6 P.M. to 8 A.M., that the postmaster and his one assistant might have ample time for eating and sleeping; when the deaths for a year in Atlanta were about one hundred and fifty; when in our unlighted streets at night the policemen, on their beats, called the passing hours with "All is well," though some house might be afire, or some citizen be dying; when our then City Fathers were gravely resolving that Messrs. Lynch and Kydd might open Whitehall street from Alabama street to the railroad track, at their own expense, and that Judge Hayden might take sand out of Marietta street, in front of the Norcross corner, "provided that for each load of sand he should return two loads of dirt."

The temptation is still stronger to talk of the trustees of 1854, all of whom are long since buried, and tell of the high positions which they occupied socially, financially, politically and otherwise. The temptation is strong to also call the roll of the members of that first faculty of the Atlanta Medical College: Thos. S. Powell, J. P. Logan, Dr. Harry Brown, the Westmorelands, John and Willis, great in medicine and surgery, H. V. M. Miller, a walking encyclopedia, and Dr. Means, the great pulpit orator, chemist and electrician. These names of trustees and faculty are "as familiar to our ears as the sound of household words," and their relatives and children are part of our population.

But this is not a meeting of the Pioneer Society of Atlanta, where such talk would be appropriate. It is at least permissible to say that the first named six of the original board of nine trustees were lawyers—showing that from the beginning lawyers took active part in encouraging and promoting this honorable institution.

Passing from these local and temporary matters, we come to the broader subject of medical education and investigation.

Of its origin, nothing is known. Men must soon have learned from experience something as to the treatment of wounds of the body and as to the alleviation of pains in the system. Moses gave us our first recognized history; but the Bible is a history of a people and a religion rather than a repository of scientific facts. Mention therein of the "healing art" is therefore only incidental. Abraham's rescue of Lot needed to be told, but how the wounded in the battle of the four kings with the five kings were healed was immaterial. Isaac's blindness made a proper backing for the story of the swindling mother and son who cheated Esau out of his birthright, but eyewashes would have marred the picture.

The hot sun of Egypt scorching the land, muddy from the annual overflow of the Nile, bred fevers and all miasmatic diseases, which dwarfed the physical statures and shortened the lives of that people. It was natural that Pharaoh, looking upon the long gray beard which swept the bosom of the patriarch, should ask Jacob, "How old art thou?" and that he should have proudly answered that he was one hundred and thirty years old, but not as old as were his ancestors. Fifteen years later Jacob died, and "Joseph commanded his servants, the physicians, to embalm his father; and the physicians embalmed Israel and forty days were fulfilled, for him; for so are fulfilled the days of those which are embalmed." About fifty years later Joseph also died, "and they embalmed him and he was put in a coffin in Egypt."

This is our first historical mention of physicians, "healers" (B. C. 1689). Their special work on the occasion of Jacob's death may have been the preservation of the cadaver, rather than saving his life. Perhaps Joseph believed in the Egyptian doctrine of metempsychosis, and therefore wished his father's body preserved as an abode to which, after wandering about in beasts and birds for three thousand years, his spirit might return. Perhaps it was a political movement to please the Egyptians, who thereupon "mourned for Jacob three score and ten days." Be that as it may, necessity made the Egyptians, in a sickly land, study medicine; physicians were a part of the household of Joseph; they surely had learned much of the medicinal properties of plants to understand embalming; it must have been long practiced to have been reduced to a specific number of days for its operation, and have been thoroughly understood to have been so perfectly done as their mummies attest. Nor was it known only to a few; for Herodotus has told us that, to protect human life, for which they had such high regard, a human being killed in any political division of the country was required to be embalmed, in the best known manner, at the expense of that community. From that possibly sprung the old English law of *deodands*, under which the thing which killed a human being was forfeited to the crown and its proceeds applied to pious uses, and the statutes by which the "hundredors," from first Edward to second George, were made responsible for violence, robbery, etc., in their "hundreds."

After the death of Joseph, Asa, the good king of Judah, reigned. He was terribly afflicted with gout, "yet in his disease he sought not to the Lord, but to the physicians," and died. The author of Second Chronicles perhaps intended to teach that Asa died because he should have sought the Lord instead of the physicians. We enter not upon that field of disputation. We may agree with King Hezekiah who, 713 years before Christ, being ill, prayed, but also took the advice of Isaiah the prophet and applied to his carbuncle a fig poultice. But the fact remains that this great and good king Asa, who deposed his mother from being queen because she had an idol in a grove, and "cut down her idol and stamped it and burnt it at the brook Kidron," when in exceeding pain from illness, had sense enough and courage enough to send for physicians trained to deal with men's afflictions. And when he was dead, they "laid him in a bed which was filled with sweet odours, and divers kinds of spices prepared by the *apothecaries'* art: and they made a very great burning for him."

It is probable that in Asa's time special hospitals or places for healing the sick had been established; for, about 885 B. C., "King Joram went back to be healed in Jezreel of the wounds which the Syrians had given him at Ramah, when he fought against Hazael, King of Syria." Let us couple that notice of surgery with that of a hundred years later, wherein Isaiah, speaking of the "wounds and bruises and putrefying sores" of Israel, said, "they have not been closed, neither bound up, neither mollified with ointment"; or notice that still two centuries later Ezekiel said, "I have broken the arm of Pharaoh, King of Egypt, and lo, it shall not be bound up to be healed, to put a roller to bind it, to make it strong to hold the sword."

But enough of medicine, pharmacy and surgery in Egypt and Palestine. Ezekiel had added to what he learned in Judea many facts gathered in his march as an exile and from observation in and about Babylon, where the sick were exposed in public streets, that they might receive not only sympathy and alms, but medical advice from all observers. Such was the clinical practice then in several nations.

How highly the Greeks esteemed the healing art may be learned from their legends as to Æsculapius; for instance, that Jove burst his skull by lightning for fear that he was about to give everlasting life to men; and from the facts that the names of two of his daughters, Panacea and Hygeia, are yet "household words," and almost everywhere we see his monumental symbols, the sacred serpents, the Agathodemons, wrapped around crockery representing the life-giving cup of Nature.

The Trojan war was about 900 B. C., but we must allow that when Homer wrote of it he spoke as one of his own century. How much of medicine the Greeks taught at Rhodes or Cos or Cnidos may not easily be known, but the Greeks, like the Egyptians, would not open or dismember the human body. Homer's knowledge of anatomy, therefore, must have been derived in some other of the countries in which he had traveled or lived. He tells us that when Menelaus was wounded, Machaon, a descendant of Æsculapius, sucked the blood from the place and applied bitter herbs thereto, and that Pæon healed the wound made in Diomedes by a brass-tipped javelin, by pouring thereon the juice of fig trees. In the Odyssey he told us how the bite of a wild boar was cured by incantation.

About three hundred and fifty years before Christ, Hippocrates won his high appellation of "The Father of Medicine." The conquests of Alexander the Great came and laid the seat of learning again upon the banks of the Nile under Greek leadership. The Alexandrian school drew to it the scholars of the world. It turned out Hierophilus and Erasistratus, whose most notable work was the discovery of the valves of the heart and the relations of the brain and nerves. Christ so spoke of physicians as to show that the people then were as familiar with the profession as with sheep and products of the farm and field, with which he so frequently illustrated his discourse: "They that are whole need not a physician, but they that are sick."

Save Galen, I recall no illustrious physician of Rome. There, until the door was opened by Pope Innocent II., the "healing art" was confined to the clergy, and in public estimation cures were worked less by science than by religion; medicine was merged in miracle.

The University of Bologna, in 1384 A.D., granted the first degree of doctor of medicine, though it had been making doctors of laws and of divinity for several centuries before. Those degrees, then and afterwards granted, carried with them many rights and privileges which we cannot bestow when we confer the degree "*cum omnibus juribus et privilegiis ei pertinentibus.*" The laws then took special notice of learned men, because they were scarce.

The laws not only gave rights and privileges to doctors of medicine, but began to protect the public against pretenders and quacks. In this regard, and indeed as to others, our citations are now confined to the laws of our mother country. In 1421, an ordinance of the city of London forbade "meddlers with physic and surgery" and would allow none to practice medicine until approved by the universities, or practice surgery until approved by masters of that art. This greater caution had doubtless been induced in part by observation when London and the nation had been so often since 1347 A.D. afflicted with the terrible plague known as the "Black death," which mowed down the population during the fourteenth and fifteenth centuries. This plague not only emptied tenements and filled ditches in the fields instead of graves, but caused the legislators, as ignorant of political economy as of the laws of health, to try to meet the consequent scarcity of labor by laws forbidding labor combinations and forcing work and sales of food at fixed prices, and the like. These acts of lords and commons, and other acts like them, were not repealed until Elizabeth, though in the teeth of the rule that prices should always be adjusted by the law of supply and demand, a law as fixed and as salutary as the law of gravitation.

In 1542 a charter was granted to "the Company of Barber-surgeons of London." These barber surgeons undertook to cure mainly by bloodletting. "Dr. Sangrado" is a fitting caricature of the whole lot of such who, disdaining the use of other means, poured hot water into and drew hot blood out of the body as a means of restoring lost health. For over three and a half centuries the then doctor's sign, a pole striped with red, has been displayed in every town, though barbers are no longer physicians; just as we continue to wear two buttons on the backs of our coats, though we no longer have sword-belts needing to be held up as once all gentlemen had, or just as we lift our hats and bend down our heads for politeness, as did Roman knights remove the horse-tail plumes, which protected their necks from swords, in token of confidence in the friendship of other knights met in the highway.

Towards the end of the fifteenth century came another plague called "sweating sickness," which at various times for sixty-five years killed as many as had fallen before the "black death"; and, from China, through India, came smallpox, which is forever renewed in all unprotected places. Lady Mary Wortley Montagu introduced from the East into England, in 1722, inoculation, but it was a preventive only a little less terrible than the disease.

In 1543 A.D. came the Apothecaries' Act; in 1620 A.D. "The Apothecaries' Company" was chartered by James I., and that business was legally separated from that of grocers.

A few facts will show the state of things between 1543 and 1620 A.D. The population of London was not much over that of Atlanta. Her citizens had begun to build chimneys to their houses instead of kindling fires on dirt in the midst of their rooms. The rich had carpets, not for floors, but for table-cloths. Table-knives were made in England in 1568, but forks came much later. Queen Elizabeth had her first silk stockings in 1562, hand-made, for the knitting-frame was not invented until 1589. She had no pins for her clothes. When she rode, a courtier sat in the saddle, and she was behind keeping her position with her arm around his waist. In the noted painting of her at Tilbury Fort, stirring up British courage to meet Spain's "Invincible Armada," she is represented as seated alone upon a charger, but he is held by men on either side of the bit, and she is mounted on what resembles the half of a thick bedtick. Speaking of horseback riding, it was not long before Elizabeth that doctors of medicine ceased to ride sideways, perhaps so riding to advertise their calling. Men wore their hats at table, that they might gracefully acknowledge compliments with appropriate bows. Their trousers were padded with bombast, that they might stuff their legs to a tasteful and graceful fatness. This was more than a hundred years ahead of sidewalks in London. In the streets it was well to walk close to the houses, to avoid the slush of the roadway on one side and the slops which might be dashed from houses overhead. For further particulars see Juvenal's satire on Rome's condition as to cleanliness, etc. See what it meant to give a lady the inside of the walk in those days.

In 1517 Erasmus had suggested, when the third visitation of the "sweating sickness" was desolating the country, that London needed drainage and sunshine, but the filthy streets still were emptied into the Thames, whence came the drinking-water of the city for centuries yet.

At last, and of course, came the climacteric affliction, the great "London plague" of A.D. 1664 and 1665. Men stood aghast before multiplied deaths, and gibbered about the evil influences of stars and planets, not fools,—but wise men, so-called. James the First had published his book on Demons in 1597. It was republished in 1603, and in that year Parliament made a long list of capital felonies which stood upon the statute books until the repeal under George the Second, the *gravamen* of which crimes was dealing with devil-possessed witches. Shakespeare was then putting upon the stage ghosts of murdered kings, and witches concocting hell-broths not unlike some medical prescriptions of his day.

Imported from France, from the time of Edward the Confessor, the sacrilegious laying on of kingly hands had been used on patients afflicted with scrofula or "King's evil." Michael Johnson was a magistrate of Litchfield, and a seller of books, so well acquainted with their contents as to be regarded as "an oracle on points of learning," and yet Lord Macaulay tells us that he in 1712 carried his son Samuel to London to be treated for scrofula, and that he was "inspected by the Court surgeon, prayed over by the Court chaplains" and "touched" by Queen Anne. His "features remained distorted," in spite of all that and of the indwelling mind which, when mature, filled so many books with his wit and learning. So William the Conqueror tried his hand about the close of the eighteenth century, dismissing his patient, however, with: "God give you better health and more sense."

The pulpit thundered out that all such plagues were scourges of Providence, and called for larger and more frequent sacrifices to the

God of the Universe, when in truth they came from gross ignorance and from too great and long-contined sacrifices to one of the deities of heathen Rome, Cloacina, the Goddess of sewers and filth.

It was in 1736 that Hogarth painted his celebrated picture of the "Consultation of the Physicians," wherein sat seven doctors of medicine, each holding a walking-stick with a hollow top filled with spices and perfumes, by smelling of which they hoped to ward off disease. To all such might justly be applied the couplet soon afterwards written by Garrick about Dr. John Hill, physician and author, viz.:

> "For physic and farces his equal there scarce is,
> His farces are physics, his physic a farce is."

We have passed in quick review from Abraham and Lot to now, a history of about three thousand and eight hundred years, one-half before and the other half since the wise men of the East followed the Star of Bethlehem.

You can place between the points of observation noted many scenes of beauty on the one hand and of horror on the other. But let us cut short this too tedious list of dates and facts, and make some practical application of them to our situation and circumstances.

The first thing suggested is how slow was and must continue to be the growth of medicine as a science. From the beginning of the fourteenth century the mariner's compass was used in Europe, and enabled men to quit the dangerous guidance of the shores or the uncertain stars for short voyages only, and sail directly, according to the pointing of the needle, to all parts of the earth. But so little was cared for human lives compared with conquest of new territory and discovery of mines, that but little was learned of botany, and but little was added to the world's "Materia Medica." Though the printing-press had been used from the middle of the fifteenth century, books of all kinds were scarce, and especially such as treated of medicine. Men had not learned to observe and reason on the subject. It is to us passing strange that though much was known about the flow of human blood, from the time of Homer down, that though a touch on the wrists told of the regular pulse-beat of the heart, it was not until A.D. 1650 that Harvey discovered the circulation of the blood, and its announcement was derided until by practice on frogs and domestic animals Malphigi, after Harvey's death, proved and also announced the certainty of the fact.

All the vitals of men are but thinly covered; so thinly that we may see the swell of heart and lungs as they perform the functions necessary for every moment of life. Yet until the latter half of the last and the beginning of this century, doctors of medicine had not learned to listen to their messages, so quick and certain when the trained ears are applied to and the practiced fingers thump upon their external coverings. What had been wanting was the acquired habit of close observation and correct reasoning from known facts. Doubtless many sneered at Jenner, observing the transmigration of birds; in summer watching the cuckoo—that thief and fraud which puts her eggs into another's nest and makes that other bring to life and feed her young—and in winter studying the suspended life of hibernating hedgehogs; but he was using and strengthening those powers which made him in 1796 watch the sores on milkmaids' hands until he proclaimed to the world that vaccination would conquer smallpox, the most frightful enemy of the human race.

Fermentation had always existed in bread and fruits and fluids, and parasites had fattened on silkworms and the like, but it remained for Pasteur and Koch and their followers in our day to discover the germ theory of disease and find cures for hydrophobia and diphtheria.

This range of observation is ever widening. Commerce girdles the globe, and from every clime brings new plants, and from them and products of earth and air chemistry concocts mixtures applicable to old and new uses. He who manufactures glasses to sweep the sky and discover new worlds above, makes those with which we see and study microbes and protoplasms and all histology. The same electricity which bores and lights our mines and tunnels, bores teeth and lights up the throat, exposes the internal secrets of the eyes, and the bones and bullets in the human body.

That body furnishes its skeleton for every doctor's office. There are mapped out all the muscles and tendons, every internal part, every artery, every vein, every nerve. Every college keeps the dead, and cuts and carves and distributes their parts for examination with as little hesitancy and with as much pleasure as we would cut and carve and distribute a turkey at dinner. With absolute knowledge of all its parts and functions, with tools and implements made of finest material and with exactest fitness for the work, the live body is opened and closed or dismembered and healed, almost without the shedding of blood and absolutely without pain, while anæsthesia holds in artificial sleep, and then antiseptics prevent suppuration or soreness from the wounds.

The press, private and professional, by types and pictures, loads the student's tables with the newest experiments and latest triumphs of science in every department of medicine, curative and preventive. The Government, which throws out banners to warn agriculture and commerce of coming storms, by its statistics shows the habitat of special diseases, the deaths from each, and the circumstances in which they occur. These statistics, gathered from the whole country, and especially from the great cities and more than half a dozen most populous States, are by each census made more accurate and reliable, and will furnish much food for thought and experiment, and do great good in the future.

You young Asclepiades, graduating here to-night, are the heirs of all the dead ages past; their accumulated wealth is yours to keep and use for the public good. That wealth will be wasted unless to it you make constant increments.

The world will not object to your use of your knowledge to gain a home for yourselves and your families. You may to a limited extent indulge in social pleasures, in politics, in literature, and the thousand things which entertain cultivated men. These are selfish considerations merely. But you must work hard at home and in exposure by day and night. And let your motto be that which Thomas Carlyle wrote above his burning candle: "*Terar dum prosim.*" (I am willing to be consumed so I may be useful.)

When you sit by gaslight, reading of the poisons and remedies which come from coal-tar, the residuum left from making gas, think of the long study and noble life of him who invented the little lamp which each coal-miner wears in his cap front, as protection against "fire-damp," and recall that when Sir Humphrey

Davy was asked why he did not have it patented, he replied that it had never occurred to him to make private gain of that which he had thus given to the world.

Your profession calls for great courage. Enlisted for life in a fight with death, let none think of you as sitting in consultation with vinaigrettes to your noses; let men feel that you fear less the diseases of the body than the disgrace of cowardice.

Admitted necessarily into the privacy of families, let every patient feel confidence in you as having taken the "Hippocratic oath" of secrecy, integrity and persŏnal purity. Many patients die from fear of telling their physicians all the truth; many of your profession are responsible for this want of trust and confidence.

Fight quackery and humbuggery as you would fight consuming fire. Superstitious follies are not all gone. Many, even educated people, yet believe in lucky and unlucky stars and days and numbers; cross themselves if they turn back on a journey; tremble if an owl hoots by night; apply mad-stones for hydrophobia; consult fortune-tellers, and believe yet in "divine healing," and even that pain and suffering are unreal creations of wicked imaginations. When Shakespeare's ghost of the murdered king of Denmark, at midnight, stalked before the guard, trembling Marcellus said to his fellow-soldier, "Thou art a scholar, speak to it, Horatio." You are scholars, and therefore it is your right and duty to speak to and of all such follies and deceptions, and drive them from the stage of action. You need not fear the denunciations of men, nor that Jove will split your skulls with lightning. The God of our salvation had as one of his specially chosen disciples, "Luke, the beloved physician."

Your opportunity is grand and glorious. They who risked their lives fighting in our late war have a Nation's thanks and admiration. Havana has been taken from the Spaniards, but who will earn thanks and admiration by rescuing it from yellow fever? In our Pacific acquisitions of territory, we approach the confines of Asia, whence came and still comes that fell destroyer cholera. What Hercules will slay that monster? Such cases need the purse of the nation as well as medical knowledge. But every-day opportunities will be to you personally abundant. Disease comes from the open houses of the poor as well as from the close rooms of the wealthy; from biting hunger and from overcrowded stomachs; "Death lurks in every passing breeze, and rides upon the storm." What will you do to stay its march? Shall consumption continue to fill one-twelfth of all the graves in our county, and your profession still admit it to be incurable? You have discovered the microbes of typhus and typhoid fevers; can you not kill the lurking devils? Shall diseases of the brain disorder the powers which make man "but little lower than the angels," and will you stand by and forever answer, like the cowardly Scotch doctor of medicine in Macbeth: "This disease is beyond my practice: . . . . I think, but dare not speak"? Consider the thousands who die in childhood; can you do nothing to stop this "slaughter of the innocents"? Let these questions be summed up in the wail of old Jeremiah: "Is there no balm in Gilead? Is there no physician there? Why then is not the health of the daughter of my people recovered?" Let the answer of yourselves and all your profession be, with loud acclaim, there is balm everywhere and physicians are everywhere, searching and applying remedies to all diseases, and the health of the people shall be recovered for the good of humanity and the glory of God.

But a word further. It is no part of my work to preach to you a sermon. But I have been talking of the human body solely from a material and scientific medical standpoint, and some may think that it has been treated too lightly. No such thing was intended. I respect the King's palace not only for its beauty and splendor, but because it holds the King, the people's sovereign. The palace may be destroyed, but "the King never dies." This tenement of clay is but the temporary home of the sovereign soul. The home may perish, but the soul is immortal. Every Christian looks for glory beyond the grave, and should cultivate the purest possible ideal of a future state. So living, we will take care of our bodies until the time comes when we will pass through life in such confidence that we may say, "Yea, though I walk through the valley of the shadow of death, I will fear no evil." For the shadow will pass away, and upon our enraptured souls will break the light of a beatific vision more glorious than human hearts can crave or imaginations conceive.

